# Optimization of Functional Toothpaste Formulation Containing Nano-Hydroxyapatite and Birch Extract for Daily Oral Care

**DOI:** 10.3390/ma16227143

**Published:** 2023-11-13

**Authors:** Alexandra-Diana Florea, Cristina Teodora Dobrota, Rahela Carpa, Csaba-Pal Racz, Gheorghe Tomoaia, Aurora Mocanu, Alexandra Avram, Olga Soritau, Lucian Cristian Pop, Maria Tomoaia-Cotisel

**Affiliations:** 1 Research Center of Physical Chemistry, Faculty of Chemistry and Chemical Engineering, Babeş-Bolyai University, 11 Arany Janos Str., 400028 Cluj-Napoca, Romania; diana_florea03@yahoo.com (A.-D.F.); cristina.dobrota@ubbcluj.ro (C.T.D.); csaba.racz@ubbcluj.ro (C.-P.R.); aurora.mocanu@ubbcluj.ro (A.M.); alexandra.avram@ubbcluj.ro (A.A.); lucian.pop@ubbcluj.ro (L.C.P.); 2 Department of Molecular Biology and Biotechnology, Faculty of Biology and Geology, Babeş-Bolyai University, 44 Republicii Str., 400015 Cluj-Napoca, Romania; rahela.carpa@ubbcluj.ro; 3 Department of Orthopedics and Traumatology, Iuliu Hatieganu University of Medicine and Pharmacy, 47 Gen. Traian Mosoiu Str., 400132 Cluj-Napoca, Romania; tomoaia2000@yahoo.com; 4 Academy of Romanian Scientists, 3 Ilfov Str., 050044 Bucharest, Romania; 5 Oncology Institute of Cluj-Napoca, 34-36 Republicii Str., 400015 Cluj-Napoca, Romania; olgasoritau@yahoo.com

**Keywords:** AFM, antibacterial activity, birch extract, enamel remineralization, nano-hydroxyapatites, oral care

## Abstract

This research work aims to develop functional toothpastes with combined enamel remineralization and antibacterial effects using nano-hydroxyapatites (nHAPs) and birch extract. Eleven toothpastes (notated as P1–P11) were designed featuring different concentrations of birch extract and a constant concentration of pure nHAPs or substituted nHAPs (HAP-5%Zn, HAP-0.23%Mg-3.9%Zn-2%Si-10%Sr, and HAP-2.5%Mg-2.9%Si-1.34%Zn). In vitro assessments involved treating artificially demineralized enamel slices and analyzing surface repair and remineralization using Atomic Force Microscopy (AFM). The Agar Disk Diffusion method was used to measure antibacterial activity against *Enterococcus faecalis*, *Escherichia coli*, *Porphyromonas gingivalis*, *Streptococcus mutans*, and *Staphylococcus aureus*. Topographic images of enamel structure and surface roughness, as well as the ability of nHAP nanoparticles to form self-assembled layers, revealed excellent restorative properties of the tested toothpastes, with enamel nanostructure normalization occurring as soon as 10 days after treatment. The outcomes highlighted enamel morphology improvements due to the toothpaste treatment also having various efficacious antibacterial effects. Promising results were obtained using P5 toothpaste, containing HAP-5%Zn (3.4%) and birch extract (1.3%), indicating notable remineralization and good antibacterial properties. This study represents a significant advancement in oral care by introducing toothpaste formulations that simultaneously promote enamel health through effective remineralization and bacterial inhibition.

## 1. Introduction

Biomaterials are intentionally designed to engage with biological entities and hold significant relevance within the realms of medical and biological pursuits, including but not limited to antibacterial interventions, regenerative medicine, and immunomodulation [1,2]. Notably, plant extracts sourced from renewable natural reservoirs have recently gained substantial attention due to their inherent attributes such as biocompatibility and therapeutic potential. These plant-derived extracts exhibit noteworthy qualities such as bioactivity, adaptability, and biodegradability. However, in contrast to synthetic materials, biomaterials derived from plant extracts face certain challenges, including inconsistencies between batches, potential allergenicity, and limitations in mechanical properties. Consequently, when endeavoring to create biomaterials based on plant extracts for medical applications, it becomes imperative to undertake comprehensive characterization, establish standardization protocols, and enforce stringent quality control measures [3].

Advanced formulations of toothpastes incorporate different active ingredients capable of providing a comprehensive oral cleansing experience while protecting the enamel surface. The chemical composition of toothpaste should be carefully determined so that the components do not affect the dental enamel and manifest their potential optimally [4]. Contemporary toothpaste formulations encompass an array of diverse active constituents which possess the capacity to facilitate a comprehensive oral hygiene regimen, concurrently safeguarding the integrity of dental enamel. The meticulous determination of toothpaste’s chemical constituents is imperative to ensure that said constituents neither compromise the structural integrity of dental enamel nor fail to fully realize their inherent therapeutic potential. The chemical composition of toothpaste typically mirrors its envisaged clinical utility, encompassing various applications, including but not limited to antibacterial, anti-inflammatory, desensitizing, remineralizing, and whitening properties [4].

Antimicrobial agents of various types have been produced to treat dental plaque formed by biofilms. Antimicrobial agents can be categorized into three groups according to the materials they are made of: organic, biological, and inorganic [5]. Nano-calcium fluoride, which reduces caries and increases labile fluoride concentration [6], silver nanoparticles (AgNPs) with antimicrobial and anti-inflammatory properties [7], and nano-hydroxyapatite (n-HAP) containing different metal ions [8,9,10] are among the inorganic compounds used in toothpaste formulation.

Because of their minimal toxicity and antibacterial action, various pharmacological features, and low cost, plant secondary metabolites have attracted the interest of many researchers as naturally produced organic antimicrobial agents [11,12]. Based on their chemical contents, many plant extracts have been shown to have significant pharmacological effects. Crude extracts and distillates, comprising phenols, alkaloids, terpenoids, glycosylated compounds, vitamins, and sterols, exhibit a wide spectrum of pharmacological activity in both in vitro and in vivo contexts [13]. Products containing these plant secondary metabolites are widely embraced for their perceived safety, health benefits, and environmental friendliness when juxtaposed with synthetic chemical additives [14].

The formulation of toothpaste is straightforward, and it remains cost-effective. Moreover, toothpaste composition can be customized using a diverse array of inorganic and organic components. Birch extract, derived from various parts of birch trees, contains a plethora of bioactive constituents, such as phenolic compounds, flavonoids, and lignans, which possess noteworthy biological properties that hold promise for applications in biomaterials [15].

Some of the pharmacological actions of the secondary metabolites in birch can be found in Table 1.

To address affected enamel healing more successfully, researchers typically combine antibacterial agents with various matrix materials to create toothpastes with varying qualities. Nanomaterials, which are attractive due to their biocompatibility, are among the most common matrix materials. Research investigations into the effectiveness of nano-hydroxyapatite (nHAP) crystals within toothpaste formulations have revealed notable enhancements in the microhardness properties of human enamel following the application of toothpaste containing nHAPs [46]. Additionally, there is evidence of the proficient occlusion of dentinal tubules by nHAP particles, which adhere to the tooth surface and impede mineral dissolution processes [47].

Synthetic stoichiometric hydroxyapatite (HAP) may contain ionic substitutions like Mg^2+^, Na^+^, and CO_3_^2−^, making it well tolerated by living tissue [48]. Also, HAP is used in toothpastes or other medical applications substituted with physiological elements which enhance its bioactivity [49].

This study investigates the utilization of four novel nanomaterials in the formulation of toothpaste products. Specifically, these toothpastes were formulated by incorporating four types of different hydroxyapatites (simple, substituted, and multi-substituted HAPs)—HAP [theoretical formula: Ca_10_(PO_4_)_6_(OH)_2_], HAP-5%Zn [theoretical formula; Ca_9.22_Zn_0.78_(PO_4_)_6_(OH)_2_], HAP-0.23%Mg-3.09%Zn-2%Si-10%Sr [theoretical formula: Ca_8.19_Mg_0.10_Zn_0.5_Sr_1.21_(PO_4_)_5.25_(SiO_4_)_0.75_(OH)_1.25_], and HAP-2.5%Mg-2.9%Si-1.34%Zn [theoretical formula: Ca_8.80_Mg_1.00_Zn_0.20_(PO_4_)_5.00_(SiO_4_)_1.00_(OH)_1.00_]. The nanostructure of the HAPs was not altered by the replacement of the elements [10,50]. The underlying advantages for the incorporation of these substitutive elements in our toothpaste formulations stems from the known ability of substitution ions to engage with adjacent tissues, thereby enhancing biomineralization and facilitating the processes associated with the regeneration of biological structures [50,51].

Zinc oxide nanoparticles exhibit antimicrobial properties against both bacteria and fungi, along with documented abilities to stimulate mineralization, cell proliferation, and differentiation [52,53].

Magnesium ions have the capacity to substitute a portion of calcium ions Ca^2+^ within the hydroxyapatite lattice. This substitution affects the crystal structure and characteristics of HAP, influencing its chemical stability, including dissolution behavior and biocompatibility. Furthermore, these ions make a significant contribution to the inhibition of acid-producing bacteria, thereby diminishing their ability to induce dental caries. Moreover, they have the capacity to ameliorate tooth sensitivity and irritation by effectively sealing exposed dentinal tubules [54].

Strontium ions can serve as a suitable substitute for calcium ions within the hydroxyapatite (HAP) structure [55]. Notably, strontium has been observed to possess desensitizing properties, which are characterized by its ability to impede or slow nerve impulse transmission within dentin [56]. Moreover, it exhibits the capability to promote remineralization, thereby reinforcing enamel and addressing the initial stages of dental decay [57].

The integration of silicon into the hydroxyapatite structure leads to an enhanced remineralization process. Additionally, it augments acid resistance without inducing any deleterious effects or irritation in the teeth or gingival tissues [58,59].

The purpose of this paper is to select and optimize the chemical composition of toothpaste formulations that possess the dual attributes of remineralization and antibacterial efficacy. To this end, we conducted a comprehensive assessment of the effectiveness of eleven distinct toothpaste formulations, each comprised of varying combinations of both pure and substituted nano-hydroxyapatite, in conjunction with birch extract. It is noteworthy that contemporary scientific literature highlights an existing gap in the realm of toothpaste development, specifically in the creation of multifaceted toothpaste products capable of delivering both remineralizing and antibacterial agents concurrently, with the potential to enhance overall enamel health. Consequently, our study represents an extension of our previous work [10], wherein we made substantial advancements by formulating specialized multisubstituted hydroxyapatites (ms-HAPs) that offer adaptability in terms of structural modifications, crystallinity adjustments, nanoparticle morphology, and size modulation [50,51]. Moreover, our formulations incorporate antibacterial properties derived from birch extract. In order to comprehensively assess the potential of these toothpaste formulations in the treatment of compromised enamel and to lay the groundwork for future investigations into novel materials for oral disease management, we conducted a rigorous examination encompassing morpho-structural characteristics, surface quality, and in vitro antibacterial activity.

## 2. Materials and Methods

### 2.1. Materials

All chemicals and reagents employed in this research were of analytical grade (AR) with a purity level equal to or exceeding 99.7%. These materials were sourced from Sigma-Aldrich and included Ca(NO_3_)_2_·4H_2_O, Mg(NO_3_)_2_·6H_2_O, Zn(NO_3_)_2_·6H_2_O, and Sr(NO_3_)_2_. Additional chemicals, namely sorbitol, polyethylene glycol (PEG 400), silicon dioxide, H_3_PO_4_, (NH_4_)_2_HPO_4,_ ammonia solution (25%), and xanthan gum, were supplied by Chempur. Tetraethyl orthosilicate (TEOS) of 98% purity was acquired from Thermo Fischer Scientific (Waltham, MA, USA) for use in experimental procedures.

Hydroglycerin alcoholic extract (96% vol. ethyl alcohol obtained from cereals, glycerin, and purified water) of *Betula verrucosa* sap (10%), made from organically harvested plants, containing 18% vol. ethyl alcohol, was purchased from Plant Extrakt, Romania.

### 2.2. Synthesis of nHAPs

The 4 types of different hydroxyapatites, simple, substituted, and two multi-substituted, were synthesized following a method developed in our labs [50,51].

### 2.3. Formulation Design and Development of Toothpastes

The four synthesized nHAPs jointly with birch extract were added in the desired toothpaste formulations, as can be seen in the next table.

The experimental design proposed three groups representing the independent variable, with 11 levels. The P1-P4 group represents the basic formulation to which varied nHAPs were added; the P5-P8 group contained the basic formulation, nHAPs (3.40%), and 1.30% birch extract; and the P9-P11 group contained variants of toothpastes without nHAPs and with varying concentrations of birch extract. As toothpaste remineralization agents, variants P1 and P5 both contained HAP-5%Zn, variants P2 and P6 contained pure nHAPs, variants P3 and P7 contained HAP-0.23%Mg-3.09%Zn-2%Si-10%Sr, and variants P4 and P8 contained HAP-2.5%Mg-2.9%Si-1.34%Zn. The experiment’s dependent variables include the toothpaste’s remineralization capacity and antibacterial action. The temperature, working technique, and reagents utilized were the standardized experimental constants.

### 2.4. Preparation of Toothpastes

To create experimental toothpaste formulations, a complex procedure involving multiple technological steps was undertaken. These steps are integral to the production of an aqueous suspension, which necessitates precise blending of various components. The initial step (Step 1) involves the mixing of a specific quantity of silica dioxide with distilled water. Following a resting period of 25 min, the suspension underwent vigorous homogenization within a sealed container, followed by an additional half hour of resting. In the subsequent step (Step 2), an aqueous suspension was prepared by dispersing hydroxyapatite in distilled water, followed by continuous mixing for a duration of 50 min. The hydrated silica dioxide was then introduced into this mixture and blended until complete homogeneity was achieved. Moving on to Step 3, sorbitol was mixed with distilled water, and subsequently, PEG 400 and xanthan gum were incorporated into the mixture. The blending continued until a fine, white paste was formed. In the final step (Step 4), the paste generated in Step 3 was thoroughly mixed with the product from Step 2, and this combined mixture was stirred for approximately 10 min, after which sodium dodecyl sulfate was added. Notably, in this step, birch extract of varying concentrations was introduced in the P5-P11 toothpaste variants. The prepared toothpaste formulations were stored at a temperature of 4 °C and were employed twice daily for the treatment of enamel slices.

### 2.5. Procedure for Obtaining Enamel Specimens

The procedure for obtaining enamel specimens adhered to rigorous ethical standards and received approval from the Ethics Committee of University of Medicine and Pharmacy, Cluj-Napoca, under Approval Number 85 dated 19 July 2017. The enamel slices were obtained using the following experimental protocol: 21 healthy third molars extracted for orthodontic purposes with healthy enamel, and without abnormalities or dental restorations on the molar surfaces, were selected and ultrasonicated in distilled water for 5 min. The apical region of the tooth was securely sealed to prevent the ingress of liquids, and the root structures were enveloped within a polymer material to enhance the manageability of the samples. Subsequently, the teeth were encased within autopolymerizing acrylate prisms (Duracryl Plus, Spofadental Inc., Jin, Czech Republic) with the coronal region remaining exposed. Two molars were used as a control (Ctrl) and were maintained in deionized water without any treatment. Nineteen molars were treated for 60 s with 37.5% orthophosphoric acid and washed for 30 s with distilled water to remove contaminants. Utilizing a microtome, specifically the Microtome IsoMet^®^, longitudinal enamel specimens measuring 7 mm × 5 mm in size and possessing a thickness of 1.5 mm were meticulously sliced from the crowns of molars. This procedure resulted in the acquisition of a total of 84 samples, which were obtained from the buccal, lingual, mesial, and distal surfaces of the 21 molars utilized in the study. The NC-negative control group (*n* = 6) was selected from the artificially demineralized slices, and each slice was immersed in deionized water. The demineralized enamel surfaces of the molars were treated with a certain toothpaste (P1–P11) according to the experimental design. The sample sizes were *n* = 6 for Ctrl, *n* = 6 for NC, and *n* = 6 for each of the eleven test groups treated with different toothpastes. 

### 2.6. Enamel Treatment with Toothpaste

Over the course of ten consecutive days, a particular toothpaste formulation chosen from the array of tested formulations (designated as P1 to P11) was administered daily to a specific experimental group of enamel slices (corresponding to P1–P11). The 10-day duration of the treatment was determined according to the remineralizing effect of nHAPs [10]. The application of this treatment involved the utilization of circular brushing motions, facilitated by a brush applicator, specifically employing the 3M^TM^ Applicator Brush, procured from Corona, CA, USA. The protocol entailed brushing the teeth for a duration of 3 min, twice a day, followed by a gentle rinse. Subsequently, the collected specimens were carefully stored in sterile containers, immersed in deionized water. Prior to conducting Atomic Force Microscopy (AFM) measurements, the specimens underwent meticulous cleaning and drying procedures.

### 2.7. AFM Investigations

Images were captured using a JEOL JSPM 4210 Scanning Probe Microscope manufactured by the Jeol Company, Tokyo, Japan in tapping mode with typical silicon nitride cantilevers [60,61,62,63,64]. After the treatment, the enamel was scanned at 5 µm × 5 µm areas. For each sample, at least five different areas were observed to acquire the Ra data (mean arithmetic surface roughness). Topographic images revealed surface morphology, while tridimensional profiles revealed Ra surface roughness [62,63]. Histograms were processed using Microcal Origin v6.0 (Microcal Software Inc., Northampton, MA, USA).

### 2.8. Evaluation of the Antibacterial Activity

The microorganisms subjected to examination within this research comprised the following: *Streptococcus mutans* ATCC 25175; *Porphyromonas gingivalis* ATCC 33277; *Enterococcus faecalis* ATCC-29212; *Escherichia coli* ATCC 25922; and *Staphylococcus aureus* ATCC 25923. The evaluation of antibacterial activity was conducted utilizing the Agar Disk Diffusion Test, a recognized method for standard antimicrobial susceptibility assessment, which entails the measurement of inhibitory zone diameters [65].

To prepare the bacterial cultures, overnight incubation in Nutrient Broth was carried out. Subsequently, suspensions were diluted to a concentration of 1% (*v*/*v*) within the culture medium. Volumes of 500 μL from these diluted suspensions were applied onto Petri plates employing sterile swabs to ensure coverage of the entire solid culture medium (Mueller–Hinton agar). In the case of *S. mutans*, an overnight culture grown in Brain Heart Infusion (BHI) at 37 °C was combined with 3.5 mL of soft Mueller–Hinton agar and uniformly poured over the plates, encircling the disks. Following incubation at 37 °C for a period of 48 h, the zones of inhibition were gauged employing a scale designed for measuring inhibition zones. All assessments were conducted in triplicate under aseptic conditions.

### 2.9. Statistical Analysis

GraphPad Prism v6.0 for Windows (GraphPad Software, Inc., La Jolla, CA, USA) was used to pursue statistical analysis following a 10-day period of enamel treatment with toothpaste formulations. All surface roughness data, denoted as Ra, represent the mean values ± standard deviation (SD) derived from a minimum of three independent experiments. To discern significant distinctions among various groups of enamel slices, statistical analysis involved the utilization of One-Way ANOVA. Also, a post-test Bonferroni’s Multiple Comparison Test was applied, with a significance level set between 0.01 and 0.001.

For the statistical evaluation of bacterial growth inhibition, the GraphPad Prism v5.0 software program was employed, employing a Two-Way ANOVA analysis supplemented with a Bonferroni posttest.

## 3. Results

### 3.1. Nanostructure of the Tested Samples

**Figure 1:**
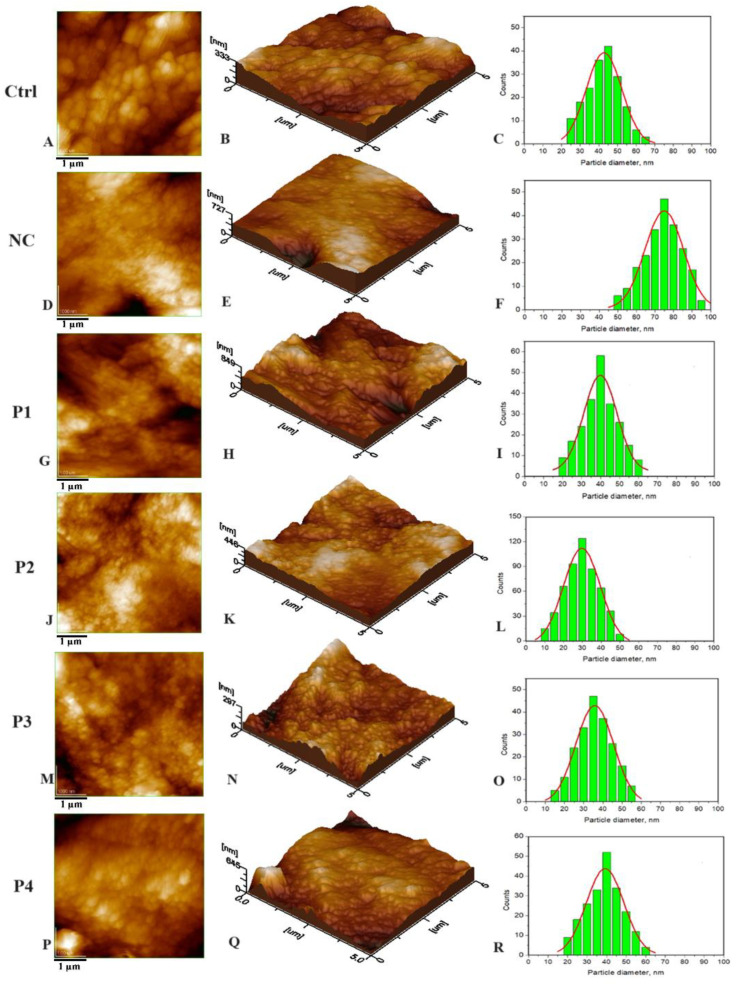
The 2D topography AFM images (**A**,**D**,**G**,**J**,**M**,**P**) and 3D topography (**B**,**E**,**H**,**K**,**N**,**Q**); scanned area of 5 µm × 5 µm. Histograms (**C**,**F**,**I**,**L**,**O**,**R**) on 2D images with the distribution of counts by particle diameter (nm) for untreated enamel (Ctrl), demineralized enamel (NC), and treated enamel with toothpastes: P1–P4.

Utilizing Atomic Force Microscopy (AFM), the dental slices, encompassing both natural specimens and those subjected to demineralization through phosphoric acid, as well as those treated with the recently formulated toothpaste, were meticulously examined. The results can be visualized in the following images (Figure 1).

By comparing the histograms of natural enamel (Ctrl) Figure 1C and artificial demineralized enamel (NC) obtained by treating samples with orthophosphoric acid (F), a significant increase in particle size was observed from values of approximately 41 nm to 75 nm.

Figure 1 also displays AFM images of HAP samples as follows: HAP-5%Zn used in toothpaste P1 (G–I), pure HAP used in P2 (J–L), HAP-0.23%Mg-3.09%Zn-2%Si-10%Sr used in toothpaste P3 (M–O), and HAP-2.5%Mg-2.9%Si-1.34%Zn used in P4 (P–R). It is important to highlight the particles observed within the hydroxyapatite (HAP) samples exhibit either spherical or oval morphology, characterized by dimensions that span from 30 nm in the case of unsubstituted HAP (as depicted in Figure 1J–L) to 40 nm for HAP containing 5% zinc (Figure 1G–I). Notably, these measurements closely resemble the size of particles found in natural enamel, which stands at 41 nm (as shown in Figure 1, labeled as Ctrl).

After a 10-day regimen of enamel treatment with toothpaste formulations, a thorough surface examination was conducted to gather data on the parameter Ra, representing surface roughness. The results unveiled substantial variations between artificially demineralized enamel (labeled as NC) and natural enamel (referred to as Ctrl).

**Figure 2:**
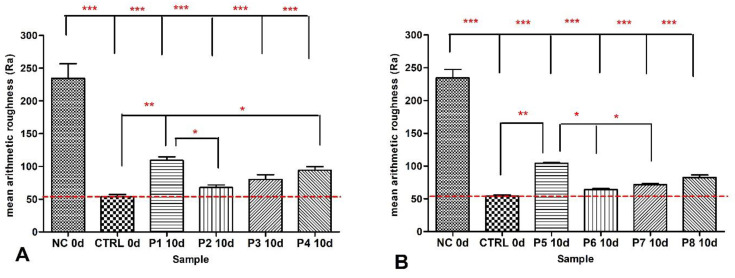
Statistical analysis of Ra values of enamel surfaces treated with toothpastes P1-P4 (**A**) compared to control (Ctrl) and demineralized enamel (NC); Ra values of enamel surfaces treated with toothpastes P5-P8 (**B**). The following degrees of statistical significance are denoted by stars: * 0.01 < *p* < 0.05; ** 0.001< *p* < 0.01; *** *p* < 0.001. Scanned area: 5 µm × 5 µm.

Following the 10-day treatment period involving toothpaste formulations P1 to P4, it was observed that the toothpaste denoted as P2, containing nanostructured hydroxyapatite (HAP), exhibited the lowest Ra values. Notably, these Ra values were not statistically distinguishable from the Ctrl values in the conducted statistical analysis, suggesting a noteworthy level of remineralization efficacy relative to the Ra value corresponding to untreated enamel. It should be noted that the Ra values for toothpaste formulations P1 to P4 exhibited a descending order as follows: P1 > P4 > P3 > P2 > Ctrl (as illustrated in Figure 2A).

The Ra values for toothpaste formulations P5–P8 exhibited the same trend of descending order: P5 > P8 > P7 > P6 > Ctrl as for toothpastes P1–P4 (Figure 2B).

### 3.2. Antibacterial Activity Evaluation

The toothpastes were inoculated in a volume of 80 µL of cultivation medium in Petri dishes. Most of the tested bacterial species are found in the oral cavity or accidentally arrive there and cause various diseases. Distilled water served as the control sample.

**Figure 3:**
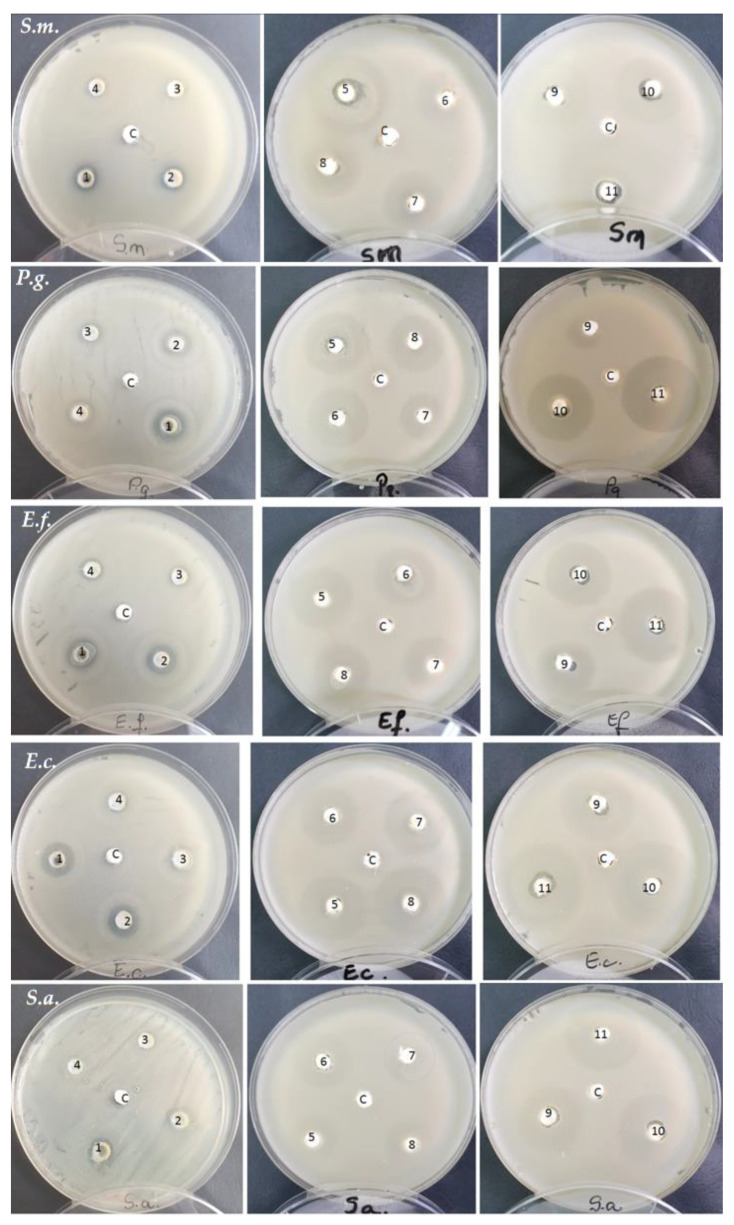
Agar disk diffusion susceptibility test of five bacterial strains *Streptococcus mutans* (*S.m*.); *Porphyromonas gingivalis* (*P.g*.); *Enterococcus faecalis* (*E.f*.); *Escherichia coli* (*E.c*.); and *Staphylococcus aureus* (*S.a*.) to the 11 toothpastes (P1–P11). The clear, circular zones around the disks indicate the growth inhibition of bacterial strains compared to control (C-distilled water).

**Figure 4:**
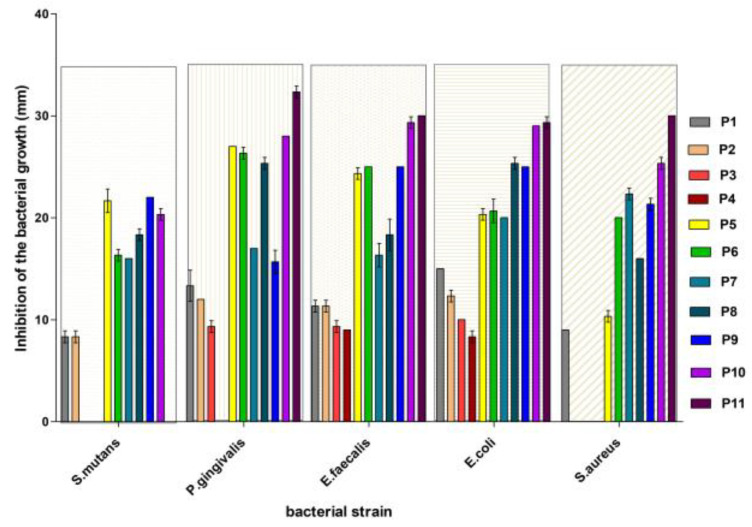
Inhibition of the bacterial growth induced by different toothpaste treatments.

The results are presented in Figure 3 and Figure 4 and Table 3.

The control sample recorded no inhibition for all strains tested in the study. *Streptococcus mutans*, a Gram-positive anaerobic coccus strain, showed resistance for samples 3 and 4 (Figure 3). P1 and P2 toothpaste samples exhibited the same growth inhibition value of 8.3 mm with a standard deviation of 0.57 mm, indicating that both toothpastes suppress the development of *Streptococcus mutans*. With a value of 22.0 mm, the P9 toothpaste sample had the maximum growth inhibition. Among all toothpastes examined, this toothpaste appears to be the most effective at suppressing the growth of *Streptococcus mutans*. With values of 21.6 mm and 20.3 mm, respectively, the P5 and P10 toothpaste samples showed considerable growth inhibition. Both toothpastes appear to be highly successful at inhibiting bacterial development, However, P9 appears to be slightly more effective (Table 3).

The diameter of the inhibitory area in the Gram-negative bacillus *Porphyromonas gingivalis* was found to be larger for samples 11, 10, 5, and 6.

For all samples analyzed, a clear region was detected in the comensal Gram-positive and anaerobic bacteria *Enterococcus faecalis*. The diameters varied, with the greatest being recorded in samples 11 and 10.

In the Gram-negative, anaerobic strain *Escherichia coli*, inhibition areas were also observed for all tested samples. In this strain, the highest inhibition diameter was obtained for samples 11 and 10. The other samples recorded smaller inhibition areas. The existence of sensitivity in the Gram-positive bacterium *Staphylococcus aureus* was shown in variants P10, P11, P9, and P7. There was no distinct region around the disks in experimental variants P2–P4. 

## 4. Discussion

This experiment on the remineralization effect of nHAPs suggests that simple HAP nanoparticles, which are smaller and uniformly arranged, have the best behavior in toothpaste due to their uniform adsorption. However, these nanoparticles must bond to the enamel structure via local epitaxial regeneration [66]. Larger nanoparticles fill deeper gaps and surround them, ensuring smoothness and optimal adsorption on uneven surfaces. This behavior can be observed in 3D imaging (Figure 1B,E,H,K,N,Q).

Figure 1A–C illustrate the fine microstructure of healthy enamel (Ctrl), which is characterized by excellent mineral material cohesiveness both inside and between the prisms. The nanoparticles are extensively welded together, resulting in a smooth enamel topography. Orthophosphoric acid etching, on the other hand, produces morphological modifications. The demineralized enamel (NC—negative control) exhibits severe surface disorganization after demineralization, as demonstrated by strongly individualizing the surrounding portions between two successive prisms and eroding the innermost layer of the prisms (Figure 1D,E). The histogram in Figure 1F (NC) shows a significant “increase” in HAP nanoparticle size from an average of 41 ± 5 nm for normal enamel (Figure 1C) to roughly 60–80 nm for etched enamel, with an average size of 75 ± 5 nm. A possible explanation for this fact can be that the demineralized nanostructural HAP unit diameter increases due to partial dissolution of the protein binder of hydroxyapatite crystallites, which tends to create some space between them [67]. The surface morphology of the treated samples indicated the presence of globular hydroxyapatite nanoparticles that exhibit uniform dispersion across the enamel surface. The average particle diameter for enamel treated with P1 was approximately 40 nm (Figure 1I), while it was around 30 nm for P2 (Figure 1L), approximately 37 nm for P3 (Figure 1O), and about 39 nm for P4 (Figure 1R). 

In Figure 2, the roughness of the surfaces expressed by mean Ra values obtained from AFM imaging is statistically analyzed. Notably, significant differences were observed between the control group (Ctrl) and artificially demineralized enamel (NC), as well as between NC and all other experimental samples (*p* < 0.001, ***). Statistically significant differences were also noted when comparing Ctrl with individual values of P2, P3, and P4 (*p* values falling between 0.01 and 0.05, denoted as *), and between Ctrl and P1 (*p* values between 0.001 and 0.01, represented as **). The relative Ra values of remineralized enamel, treated with toothpaste, exhibited a descending order as follows: P1 > P4 > P3 > P2 > Ctrl, with close values noted between Ctrl and P2, as well as P3. It is pertinent to mention that the effectiveness of these toothpaste formulations in the process of remineralizing artificially demineralized enamel could be ranked in the following order: Ctrl = P2 = P3 > P4 > P1. These claims are supported by the fact that small-sized HAP nanoparticles are capable of penetrating the pores and cracks of the dental enamel and repairing the damaged microstructure of the tooth. During the restoration of the demineralized enamel, synergy among the substituted ions released from the ms-HAPs might be observed. From the literature, it is known that substituted ions can interact with protein units to enhance the enamel resistance [68,69].

In the context of enamel surface remineralization induced by the treatment with the toothpastes denoted as P5–P8, containing HAPs and birch extract (as given in Figure 2B), a similar trend was observed as in the case of toothpaste formulations (P1–P4), which exclusively comprised only HAPs. Consequently, it can be inferred that the incorporation of birch extract does not exert a discernible influence on the remineralization potential of HAPs (unpublished data).

After testing the antimicrobial activity of toothpastes, the following observation can be drawn: namely, all strains showed varying sensitivity for each tested sample. Analyzing the data within the P1–P4 group represented by toothpastes containing only nHAPs, it can be found that there is some antibacterial activity, including in the P2 toothpaste containing only pure nHAPs. Of the first group of tested toothpaste, P1 and P2 were more effective at stopping the growth of bacteria in the culture medium. Within this group, the most significant inhibition, manifested in most species of bacteria, was recorded in the experimental variant P1 consisting of nHAP-5%Zn. It is known from the literature that zinc has an antimicrobial effect [70,71]. 

In the group of P5–P8 toothpastes containing both nHAPs (3.4%) and 1.3% birch extract, there was a great variability in the inhibition reaction of bacterial strains depending on the species. In general, P5, represented by nHAP-5%Zn, induced the most pronounced inhibition within the group in all bacterial species except *Staphylococcus aureus* (Table 3). It should be noticed that the P7 variant, represented by HAP-0.23%Mg-3.09%Zn-2%Si-10%Sr, significantly inhibited this species.

Comparing the P1–P4 group that contained only nHAPs with the P5–P8 group where birch extract was added, significant differences were found in terms of inhibition of the growth of bacterial strains. This inhibition can be attributed to the secondary metabolites contained in the birch extract. A particular case is *Staphylococcus aureus*, which showed resistance to the P2–P4 variants of toothpaste but whose growth was inhibited in the presence of birch extract in P6–P8 toothpastes. Similarly, *Streptococcus mutans* was resistant to P3–P4 variants but was inhibited by the addition of birch extract in P5–P8 toothpastes.

In the next experimental group (P9–P11) without nHAPs, the concentration of birch extract added to toothpaste was reduced (Table 2). The analysis of the P9–P11 group reveals that there was significantly greater inhibition of bacterial growth compared to the previous toothpastes, especially at 1.3% birch extract concentration. 

Among the examined bacterial species, *Enterococcus faecalis* and *Escherichia coli* had the greatest sensitivity, with *Porphyromonas gingivalis* close behind. *Streptococcus mutans* was classified in the center of the bacterial species tested. Among the examined species, *Staphylococcus aureus* had the lowest growth inhibition under the applied treatments.

Overall, based on the results from the experiments performed under the same conditions, the toothpastes can be ranked in terms of growth inhibition effectiveness (Table 3) for each bacterial species (from the highest to the lowest) as follows:

For *Streptococcus mutans*: P9 > P5 > P10 > P8 ≈ P7 ≈ P6 > P1 ≈ P2 > P3 ≈ P4 ≈ P11.
For *Porphyromonas gingivalis*: P5 > P6 > P11 > P10 > P8 > P7 ≈ P9 > P1 > P2 > P3 > P4.
For *Enterococcus faecalis*: P11 > P10 > P9 = P6 ≈ P5 > P8 > P1 > P2 > P3 > P4.
For *Escherichia coli:* P11> P10 > P8 ≈ P9 > P5 = P6 ≈ P7 > P1 > P2 > P3 > P4.
For *Staphylococcus aureus*: P11 > P10 > P8 ≈ P9 > P5 = P6 ≈ P7 > P1 > P2 = P3 = P4.

The efficiency of toothpaste varies depending on the bacterial species. Toothpaste may be extremely efficient against one species while being ineffective against another. Toothpaste P11, consisting of a basic formulation with 1.3% birch extract, appears to be among the most effective at inhibiting growth in all studied bacterial species. Toothpaste P10 is frequently listed as one of the most effective, but its efficacy differs according to species. P5 and P6 toothpastes are effective against several species, most notably *Porphyromonas gingivalis*. Toothpastes P1–P4, while inhibiting in some cases, are generally less effective than others.

According to these data, toothpaste P11 is the most effective against bacterial strains, ranking first in three of the five bacterial species studied. It consistently outperformed other bacterial inhibitors across a wide range of bacterial species, making it the clear choice for bacterial growth inhibition. 

Considering both remineralization effectiveness, expressed by the ranking Ctrl ≈ P6 ≈ P2 > P7 ≈ P3 > P8 ≈ P4 > P5 ≈ P1, and antibacterial effectiveness, expressed by the ranking P11 > P10 > P9 > P5 > P6> P8 > P7 > P1 > P2 > P3 > P4, it can be identified that toothpaste P5 is an appropriate option for both effective remineralization and antibacterial activity. This shows that toothpaste P5 may provide an efficient combination of remineralization and antibacterial properties.

As future developments of this experiment, time-course tests can be conducted to measure the lifetime of toothpaste’s efficiency in preventing bacterial growth over different time intervals. In addition to growth inhibition trials, oral microbiome studies may shed light on the broader effects of toothpaste variants on the oral microflora. Exploring other oral health parameters, such as the anti-inflammatory or anti-adhesive properties of toothpastes and investigating the mechanisms by which specific toothpaste variants inhibit bacterial growth, could provide insights for developing targeted oral care products. By integrating these future discoveries, we can acquire a better understanding of toothpaste effectiveness and enhance oral health outcomes. These improvements can contribute to the development of more effective oral care products in addition to enhancing oral hygiene practices.

## 5. Conclusions

Oral healthcare holds considerable significance in the broader context of overall well-being and quality of life. There is an increasing trend toward the adoption of functional toothpaste formulations for routine oral health maintenance. The goal of this research paper is to formulate functional toothpaste products that possess the dual capabilities of enamel remineralization and antibacterial properties, achieved through the incorporation of nano-hydroxyapatite and birch extract.

When considering both remineralization and antibacterial effectiveness, toothpaste P5 emerged as a compelling option due to its moderate antibacterial activity and good remineralization potential.

Optimizing the chemical composition of a functional toothpaste by incorporating hydroxyapatite and birch extract is a challenging process. The substantial enhancement in remineralization (P1–P4) attributed to nano-hydroxyapatite is intricately regulated by the presence of substituted elements. It is worth noting that the simultaneous substitution of multiple ions imparts a superior influence compared to the substitution of a single ion, indicating a synergistic interaction among these ions. Furthermore, it is noteworthy that the inclusion of birch extract does not alter the remineralization potential of HAPs.

In terms of antimicrobial activity, the most robust effectiveness of toothpastes was observed in formulations (P9–P11) devoid of nHAPs, as the presence of nHAPs (P5–P8) mitigated the immediate impact of birch extract. It would be of scientific interest to elucidate the mechanism underlying this mitigation of birch extract’s effect by the presence of nHAPs, which could involve a delay in the release of active molecules or a reduction in their efficacy.

## Figures and Tables

**Table 1:** Pharmacological activity of birch secondary metabolites.

Plant Part	Chemical Constituents	Pharmacological Effects	References
Roots	Essential oils, ascorbic acid, coumarins, sterols, saponins, tannins, potassium, sodium	Antiscorbutic, antiarthritic, anticancer, antidiabetic, anti-inflammatory, antimicrobial, antioxidant, antiviral, gastroprotective, hepatoprotective, immunomodulatory	[15,16,17,18,19,20,21,22]
Outer bark	Pentacyclic triterpenes (mainly betulin up to 34%) terpenes, methylsalicylate, creosol, guaiacol	Antibacterial effect against *Streptococcus*, *Porphyromonas gingivalis, Streptococcus pyogenes, Escherichia coli, Staphylococcus aureus*, and *Enterococcus faecalis*; and antifungal, anticarcinogenic, antiperiodontic, anticancer, anti-inflammatory, antiviral, antibiofilm activities.	[17,18,23,24,25,26,27,28,29,30,31,32,33]
Bark extracts	Phenols, terpenoids, alkaloids, glycosylated molecules, organic acids, cathecol, oleuro-pein-aglycone, acacetin, kaempferide, dimethylquercetin, pentacosyl, resorcinol	Anti-inflammatory, antimicrobial, antioxidant, antiviral, immunomodulatory, antiarthritic, anticancer, antidiabetic, gastroprotective, hepatoprotective, prevention of degenerative diseases.	[16,23,34,35,36]
Birch buds	Essential oil (up to 3.8%) triterpenoids, diarylheptanoids, phenylbutanoids, lignans, phenolics and flavonoids Sesquiterpene	Antiarthritic, anticancer, antidiabetic, anti-inflammatory, antimicrobial, antioxidant, antiviral, gastroprotective, hepatoprotective, and immunomodulatory activities.	[23,37,38,39]
Leaves	Flavones glycosides (1–3%), quercetin, glycosides, kaempferol glycosides myricetin glycoside, betulorentic acid, caffeic acid, saponins, tannins, sesquiterpenes, chlorogenic acid, triterpene alcohol, malonyl esters of dimarene type, polymeric proanthocyanidins, macro- and micronutrients up to 38%	Oral health as preventive and therapeutic agent, anti-inflammatory antioxidant, bactericidal effect, natural surfactants.	[17,18,40,41,42,43]
Birch sap	Al, Ca, Mg, Zn, and Ni, ascorbic malic, citric, phosphoric, and succinic acids, botulin, betulic acid	Antiscorbutic, anticancer, bacteriostatic, anti-inflammatory.	[19,20,21,22,31,32,44,45]

**Table 2:** Chemical formulations of the toothpastes.

Variant	nHAP 3.40% for Each Formulation	Birch Extract (%)	Basic Chemical Composition of the Group (%)	
P1	HAP-5%Zn	-	Distillated water	58.58
			Glycerol	25.18
P2	Pure HAP	-	Sorbitol	2.99
			Silicon dioxide	3.73
P3	HAP-0.23%Mg-3.09%Zn-2%Si-10%Sr	-	Xanthan gum	0.17
			Na lauryl sulphate	0.17
P4	HAP-2.5%Mg-2.9%Si-1.34%Zn	-		
P5	HAP-5%Zn	1.30	Distillated water	58.49
			Glycerol	25.15
P6	Pure HAP	1.30	Sorbitol	2.99
			Silicon dioxide	3.73
P7	HAP-0.23%Mg-3.09%Zn-2%Si-10%Sr	1.30	HAP	3.42
			Xanthan gum	0.17
P8	HAP-2.5%Mg-2.9%Si-1.34%Zn	1.30	Na lauryl sulphate Ethyl alcohol	0.17 3.7
			Distillated water	83.32
			Glycerol	5.9
			Silicon dioxide	5.32
P9	-	0.25	Xanthan gum	0.25
			Na lauryl sulphate	0.25
			Sorbitol	4.27
			Ethyl alcohol	0.44
			Distillated water	79.41
			Glycerol	9.06
			Sorbitol	4.06
P10	-	0.70	Silicon dioxide	5.06
			Xanthan gum	0.23
			Na lauryl sulphate	0.23
			Ethyl alcohol	1.25
			Distillated water	74.2
			Glycerol	13.15
			Sorbitol	3.8
P11	-	1.30	Silicon dioxide	4.72
			Xanthan gum	0.21
			Na lauryl sulphate	0.21
			Ethyl alcohol	2.41

**Table 3:** Bacterial growth inhibition induced by toothpastes.

	Diameter of the Inhibition Zone (mm)				
Sample	*Streptococcus mutans*	*Porphyromonas gingivalis*	*Enterococcus faecalis*	*Escherichia coli*	*Staphylococcus aureus*
P1	8.3 ± 0.57	13.3 ± 1.52	11.3 ± 0.57	15.0 ± 0	9.0 ± 0
P2	8.3 ± 1.15	12.0 ± 0	11.3 ± 1.15	12.3 ± 0.57	-
P3	-	9.3 ± 0.57	9.3 ± 0.57	10.0 ± 0	-
P4	-	0	9.0 ± 0	8.3 ± 1.52	-
P5	21.6 ± 1.15	27.0 ± 0	24.3 ± 0.57	20.3 ± 0.57	10.3 ± 0.57
P6	16.3 ± 0.57	26.6 ± 1.15	25.0 ± 0	20.3 ± 1.15	20.0 ± 0
P7	16.0 ± 0	17.0 ± 0	15.3 ± 1.15	20.0 ± 0	22.3 ± 1.15
P8	18.3 ± 1.52	25.3 ± 0.57	18.3 ± 1.52	25.3 ± 1.15	16.0 ± 0
P9	22.0 ± 0	15.3 ± 1.15	25.0 ± 0	25.0 ± 0	21.3 ± 1.52
P10	20.3 ± 0.57	28.0 ± 0	29.3 ± 0.57	29.0 ± 0	25.3 ± 0.57
P11	-	32.3 ± 0.57	30.0 ± 0	29.3 ± 0.57	30.0 ± 0

## Data Availability

Data are contained within the article.
